# AR Bs and possible cancer risk

**Published:** 2010-08

**Authors:** J Aalbers

**Affiliations:** Special Assignments Editor

## Introduction

The recent meta-analysis published in the Lancet Oncology, with comment from Dr Steve Nissen,^1^,^2^ has raised questions around a possible cancer risk and the use of ARBs. The Cardiovascular Journal of Africa will be publishing a fuller report on this matter in a forthcoming issue of the Journal.

In the interim, Boehringer-Ingelheim has issued the following pertinent comments.

## Comment from Boehringer Ingelheim

Boehringer Ingelheim affirms safety of telmisartan with an analysis of 50 000 patients and strongly disagrees with the publication of Sipahi et al.^1^ in Lancet Oncology June 2010

Telmisartan, an angiotensin receptor blocker, is one of the best-researched drugs worldwide. It has been studied in clinical trials in more than 50 000 patients. Its positive safety profile has been confirmed also in a market exposure of 34.5 million patient years.

Convincing safety data for patients with a high cardiovascular risk were collected in the three long-term outcome trials ONTARGET, PRoFESS and TRANSCEND, which followed some of the patients for up to five years. Following rigorous assessment of the data from these studies it was concluded that there was no association with an increased risk of cancer in the telmisartan arms.

Sipahi et al.^1^ published a meta-analysis in the June issue of Lancet Oncology, claiming that angiotensin receptor blockers (ARBs) used to lower hypertension are associated with a modestly increased risk of new cancer diagnosis. The finding is mainly based on the combination arm of telmisartan and ramipril, an angiotensin converting enzyme (ACE) inhibitor, and not on the trial arms of each compound separately.

Patient health and safety is the primary concern of Boehringer Ingelheim. The company continually monitors safety data for all medical products. Boehringer Ingelheim’s comprehensive internal safety data analysis of primary data contradicts the conclusions about an increased risk of potential malignancies mentioned by Sipahi et al.^1^

All studies with telmisartan included patients with cardiovascular risk factors due to age and co-morbidities. Specifically, in ONTARGET, with more than 25 000 patients, no statistically significant difference with respect to malignancies was observed in patients treated with telmisartan vs ramipril. In TRANSCEND, a 6 000 patient trial, the difference did not reach significance either. In the PRoFESS trial, another large-scale trial with more than 20 000 patients, the telmisartan arm showed fewer cases of malignancies than the placebo arm. Considering the analysis of all three trials, an effect of telmisartan on malignancies was not observed.

In ONTARGET, the one treatment arm with a combination of telmisartan and ramipril was associated with a modestly increased risk of malignancies. Consistent with our commitment to transparency, data from ONTARGET, TRANSCEND and PRoFESS have all been published and been widely shared with regulatory authorities since 2008. It should be noted that product labelling for telmisartan does not recommend the combination of telmisartan and ACE inhibitors such as ramipril.

‘Our research efforts have centred on the need to protect patients, especially older patients, from cardiovascular risks such as myocardial infarction or stroke. Telmisartan fulfills this need. It is the only ARB that has cardiovascular protection in its label and has become a valuable treatment option in the management of hypertension and cardiovascular risk. Doctors and patients appreciate its excellent safety profile.

In pre-clinical trials, clinical trials and day-to-day patient exposure with telmisartan, we have not seen any significant finding related to malignancies. Patients should consult with their physicians before making any decision regarding their antihypertensive therapy’, said Prof Dr Klaus Dugi, Corporate Senior Vice President, Medicine at Boehringer Ingelheim.

Peer-reviewed meta-analyses of aggregate published data like Sipahi et al.^1^ have their appropriate place in scientific research. However, these analyses have well-recognised limitations, such as combining study summaries rather than analysing individual patient data.

Telmisartan is one of the most studied antihypertensives in clinical trials, which have all been made publically available. It is widely used as medication to lower blood pressure and protect patients against severe cardiovascular events such as myocardial infarction and stroke.

Please be advised: In some countries Micardis has not yet been registered for the cardiovascular protection indication. Please refer to the package insert approved by the local regulatory authority.

S3 Micardis® 40 and 80 mg. Each tablet contains telmisartan 40 and 80 mg, respectively.

S3 Pritor® 40 and 80 mg. Each tablet contains telmisartan 40 and 80 mg, respectively.

For further information please contact: Dr Kevin Ho, medical director: Tel: + 27 11 348-2517; e-mail: kevin.ho@boehringer-ingelheim.com

Sue Thomas, medical information manager: Tel: + 27 11 348-2514; e-mail: sue.thomas@boehringer-ingelheim.com

## References

[1] Sipahi I, Debanne SM, Rowland DY, Simon DI, Fang J Angiotensin-receptor blockade and risk of cancer: meta-analysis of randomised controlled trials..

[2] Nissen SE

